# Applying Precision Medicine to the Heterogeneity of Asthma Attacks

**DOI:** 10.1016/j.chest.2025.11.013

**Published:** 2025-11-13

**Authors:** Carlos Andres Celis-Preciado, Elsa Ben Hamou-Kuijpers, Sanjay Ramakrishnan, Imran Howell, Michael E. Wechsler, Praveen Akuthota, Simon Couillard

**Affiliations:** a Faculté de Médecine et des Sciences de la Santé, Université de Sherbrooke, Sherbrooke, QC, Canada; b Internal Medicine-Pulmonary Unit, Hospital Universitario San Ignacio, Bogota, Colombia; c Institute for Respiratory Health, University of Western Australia, Perth, WA, Australia; d Nuffield Department of Medicine, University of Oxford, Oxford, England; e Division of Pulmonary, Critical Care and Sleep Medicine, National Jewish Health, Denver, CO; f Division of Pulmonary, Critical Care, and Sleep Medicine, Department of Medicine, University of California, San Diego, San Diego, CA

**Keywords:** asthma, attacks, biomarkers, corticosteroids, endotypes, eosinophils, etiology, Feno, heterogeneity, phenotypes, precision medicine

## Abstract

**Topic Importance:**

The standard of care for management of asthma attacks has remained unchanged for 70 years, relying on a symptom-based, severity-stratified approach. Severe asthma attacks are defined by a worsening of asthma requiring oral corticosteroid (OCS) treatment for unresolved symptoms for at least 48 hours, decreased lung function, or both. The 1-size-fits-all strategy with OCS treatment overlooks the biological mechanisms driving attacks and may lead to suboptimal outcomes. Importantly, OCS-related toxicities lead to significant morbidity, and cumulative OCS use has been associated with increased mortality. Antibiotics, often used indiscriminately, also increase adverse events and antimicrobial resistance.

**Review Findings:**

Recent studies have highlighted the heterogeneity of asthma attacks across clinical, etiologic, and therapeutic dimensions. Biomarker-informed assessments using blood eosinophils, exhaled nitric oxide (Feno), and point-of-care microbial molecular testing have improved the evaluation of attacks. Observational studies and trials have explored biomarker-guided management to reduce OCS and antibiotic use, potentially improving outcomes. Distinct inflammatory and OCS response profiles were identified in patients receiving biologics, emphasizing the complexity of attacks and the importance of residual (untreated) type 2 inflammatory pathways. Studies of the airway microbiome revealed that microbial dysbiosis is associated with clinical and inflammatory clusters.

**Summary:**

Asthma attacks are complex episodes with diverse causes, endotypes, and phenotypes. Emerging evidence supports incorporating biomarkers (blood eosinophils, Feno, and microbial testing) into clinical assessment to refine management. Recent evidence expands our understanding of exacerbation mechanisms, highlighting the need for tailored management strategies. Recognizing asthma heterogeneity could shift care toward precision medicine, reducing OCS reliance and improving patient outcomes.

Asthma is a chronic respiratory disease affecting 400 million people in the world. It is defined by the presence of respiratory symptoms, bronchial inflammation, airway hyperresponsiveness, and remodeling.^1^ Exacerbations, also called attacks, are a hallmark of the disease.^1^^,^^2^ Asthma attacks are major contributors to the disease burden. These events are unpredictable, yet they predict the risk of subsequent attacks,^3^ creating a vicious cycle defining disease severity.^1^ Over time, attacks contribute to a progressive decline in lung function^4^ and worsening quality of life.^5^ Oral corticosteroid (OCS) therapy used to treat attacks also increase the risk of comorbidities developing, including cardiovascular disease, osteoporosis, diabetes, and obesity, collectively referred to as people remodelling.^6^^,^^7^ Intermittent OCS exposure also is associated with health care resource use and is linked to a higher risk of all-cause mortality.^8^, ^9^, ^10^ Although not recommended routinely, antibiotics are used commonly in clinical practice,^11^ increasing antimicrobial resistance and adverse effects. This review explores asthma exacerbation heterogeneity, focusing on limitations in definitions, severity stratification, clinical phenotypes, triggers, endotypes, and microbiome. It also discusses current and emerging management strategies, including precision medicine in acute asthma.

## Literature Search

A literature search was conducted in MEDLINE for articles about asthma attacks published through December 1, 2024, using the terms (*exacerbation* OR *attack* OR *flare* AND *asthma* [Medical Subject Headings]). Titles and abstracts were reviewed, and relevant full-text articles were assessed. Additional studies were included manually to highlight recent information about asthma attacks in adults.

## Evidence Review

### Definition of Asthma Attacks

Asthma attacks are defined as episodes with a progressive increase in respiratory symptoms, often coupled with a decline in lung function, and requiring treatment adjustments.^2^ The Global Initiative for Asthma (GINA) guidelines recommend OCS treatment for acute asthma marked by a peak expiratory flow or FEV_1_ of < 60% of the patient’s personal best or predicted value, or a lack of response to increased inhaled treatment use over 48 hours.^1^ Systemic corticosteroid use, emergency room visit, hospitalization, or a combination thereof qualify the attack as severe.^1^^,^^12^

This definition has several limitations that may affect its clinical applicability and accuracy. Indeed, a recent subanalysis of asthma attacks in the Clinical Study in Asthma Patients Receiving Triple Therapy in a Single Inhaler (CAPTAIN) study, a multicenter, randomized controlled trial evaluating dual vs triple inhaled therapy in asthma, highlighted several key considerations.^13^ One major finding was the overlap in severity indicators between moderate and severe attacks, because both categories exhibited similar lung function decline, increased symptoms, and frequent reliever use. This overlap challenges the rigid classification, suggesting that attacks exist on a continuum, rather than as distinct categories.^13^ Another critical finding involved reliance on patient-reported symptoms that can be inconsistent because of variability in symptom perception and reporting accuracy.^14^ Finally, the CAPTAIN study emphasized the relevance of inflammatory biomarkers such as blood eosinophils and exhaled nitric oxide (Feno) to distinguish attacks and potentially to guide individualized treatments.^15^, ^16^, ^17^ This dimension is notably absent from traditional definitions of asthma attacks.

### Heterogeneity of Clinical Presentation: Phenotypes

Asthma attacks are variable in their clinical presentation, reflecting the complex interplay among triggers, underlying inflammatory mechanisms, and patient-specific factors. Two broad clinical phenotypes have been defined: slow-onset and fast-onset attacks.^18^^,^^19^ Slow-onset asthma attacks typically develop over hours to days and constitute 80% to 90% cases. They usually are triggered by viral respiratory infections and can exhibit neutrophilic or eosinophilic inflammation.^20^^,^^21^ Patients generally demonstrate less severe symptoms on arrival, but are less responsive to initial treatment, and these patients are associated with prolonged hospital stays.^18^^,^^19^ Fast-onset asthma attacks are less common (< 20% of cases), and patients seek treatment within a few hours (< 3 hours) of symptom onset. These attacks often are precipitated by identifiable triggers (allergens, irritants, nonsteroidal antiinflammatory drugs, or stress). Attacks tend to involve bronchial neutrophilic inflammation, rather than eosinophilic inflammation, and are characterized by more severe clinical features at presentation, necessitating urgent medical care.^22^ However, they show a larger improvement after initial treatment, and hospital stays tend to be shorter.^18^^,^^19^

Although clinical phenotyping may provide insights into different trajectories, it does not inform underlying pathobiological features. Consequently, this classification does not allow precise mechanistic targeting of therapies. A deeper understanding of the biological drivers—through biomarker profiling—is required to guide personalized treatment decisions beyond clinical presentation alone.

### Heterogeneity of Pathobiology: Etiotypes and Endotypes

Although often it is difficult to identify a precipitating cause of exacerbations, asthma attacks are triggered by a diverse array of stimuli, including viruses, bacteria, allergens, and pollutants. Despite similar clinical presentations, the mechanisms associated with these provocations often are distinct.

#### Common Triggers: Interplay and Overlap

##### Viral Infections

Respiratory viruses, led by rhinovirus, drive 47% of asthma attacks, as shown in a systematic review of 14 studies encompassing 1,265 adults.^23^ Approximately 10% of patients harbored multiple viruses.^24^ Mechanistically, translational studies have shown reduced production of interferons by airway epithelial and dendritic cells in patients with asthma. Both type 2-high and type 2-low asthma exhibit reduced interferon responses, correlating with infection severity and increasing asthma attack rates.^25^^,^^26^ In children, viral infections are more predominant in driving asthma attacks, with prevalence as high as 72% of patients.^24^

##### Type 2 Inflammation

This immune response is characterized by the actions of key cytokines IL-4, IL-5, and IL-13. Although the response may be initiated by epithelial alarmins (thymic stromal lymphopoietin, IL-33) through the activity of Th2 and Group 2 innate lymphoid cells, it is mediated by eosinophils and other effector cells.^27^ Over the past 2 decades, it has become clear through autopsy studies^28^ and analyses of large international trials’ control arms^16^^,^^17^^,^^29^^,^^30^ that type 2 inflammation characterizes fatal attacks, also predicting increased rate of severe events.

##### Bacterial Infections

A systematic review analyzing 43 studies (3,511 adults) found bacterial pathogens in 7% of patients with asthma attacks, with *Haemophilus influenzae* (2%) and *Streptococcus pneumoniae* (2%) being common culprits, alongside atypical pathogens like *Chlamydia pneumoniae* (4%) and *Mycoplasma pneumoniae* (4%).^24^ Mechanistically, bacteria activate toll-like receptors on dendritic cells, shifting immune responses from type 2 to type 17 pathways and the recruitment of neutrophils that produce neutrophil extracellular traps, amplifying tissue damage.^31^

##### Allergens

Environmental allergens significantly contribute to asthma attacks because of widespread exposure and high sensitization rates (> 80% in children). A systematic review linked total, grass, and weed pollen concentrations to severe attacks.^32^ Sensitization to allergens like fungal spores (eg, *Alternaria alternata*) heightens the risk of emergency care and admission to intensive care.^33^ Pollen-related thunderstorm asthma, triggered by ryegrass pollen and fungal spores, underscores the interplay of environmental and allergic factors.^34^ Allergic sensitization further increases attack risk when combined with respiratory viral infections.^35^ In a human model of allergen-induced asthma exacerbation using segmental allergen challenge and comparing patients with allergic asthma and with patients without asthma showed that unique to the asthmatic group were IL-9-expressing pathogenic type 2 cells that upregulated genes sustaining type 2 inflammation.^36^

##### Pollutants

*Pollutants*, both outdoor and indoor, increase asthma exacerbation risk. Short-term exposure to particulate matter (fine particulate matter ≤ 2.5 μm and ≤ 10 μm in diameter) and nitrogen dioxide raises the risk of emergency visits and hospitalizations.^37^ Systematic reviews have shown that factors like heavy traffic, fumigants, and extreme weather also worsen asthma, although with varying certainty.^38^, ^39^, ^40^, ^41^ Mechanistically, air pollutants can activate both type 2 and non-type 2 inflammatory pathways involved in asthma attacks.^42^

#### Inflammatory Endotypes of Asthma Attacks

##### Biologic-Naive Asthma

Studies measuring inflammation during asthma attacks collectively have underscored the heterogeneity of sputum inflammatory responses across attack severities.^43^, ^44^, ^45^, ^46^ The Phenotyping the Responses to Systemic Corticosteroids in the Management of Asthma Attacks (PRISMA) study by Celis-Preciado et al^47^ phenotyped 53 severe asthma attacks of all disease severities before and after OCS use in biologic-naive patients. Attacks were classified according to an ordinal blood eosinophil count (BEC)-Feno 3-group categorization (type 2-low/low: BEC < 150 cells/μL and Feno < 25 parts per billion [ppb]; type 2-high/high: BEC ≥ 300 cells/μL and Feno ≥ 35 ppb; and type 2-mid: not meeting low/low-high/high criteria). Sixteen (30%) type 2-low/low, 27 (51%) type 2-mid, and 10 (19%) type 2-high/high attacks were included in that study. Participants moving from type 2-low/low to type 2-high/high categories were significantly more likely to be male and to have greater prevalence of nasal polyposis, previous history of type 2-high status in the past 12 months, FEV_1_ reversibility after bronchodilation, and lower FEV_1_ to FVC ratio values after bronchodilation. In contrast, lower type 2 biomarkers were associated with significantly higher Nijmegen questionnaire scores, suggesting a possible contribution of dysfunctional breathing patterns to symptom burden in this subgroup. Notably, no differences between groups were found regarding GINA treatment step, symptom scores, FEV_1_ after bronchodilation, the delta of FEV_1_ after bronchodilation compared with the best value from the previous 12 months, chest radiograph findings, frequency of viral or bacterial infections, or use of antibiotics. These findings suggest that BEC and Feno stratify attacks independently of the presence of clinical features, confirming their complementary mechanistic value.^48^ Biomarker-stratified outcomes after OCS treatment in the PRISMA study are discussed further in the section entitled Emerging Strategies: Precision Medicine in Action.

McDowell et al^49^ studied 71 attacks in the Refractory Asthma Stratification Program study. Events were categorized as type 2-low (BEC < 150 cells/μL and Feno < 20 ppb) or type 2-high. In this population with severe asthma, 19 attacks (27%) were type 2-low. Neither an increase in symptoms nor a reduction in lung function from the clinically stable state enabled the distinction between the inflammatory phenotypes. Interestingly, the phenotype in the stable state was not associated significantly with the phenotype during exacerbation.

A study by Ghebre et al,^50^ published in 2018, comprehensively investigated the inflammatory phenotypes of the attacks of 32 patients with asthma and 73 patients with COPD using a cross-sectional design, multimodal clinical assessment, measurements of sputum cell counts, cytokine concentrations, and microbiome analysis. Three clusters were identified. Cluster 1 was characterized predominantly by neutrophilic inflammation in blood and sputum and higher proportions of bacteria-associated attacks. Cluster 2 included patients with an eosinophilic-predominant inflammation in sputum and blood. Cluster 3 was associated primarily with viral-induced inflammation, with elevated sputum and serum levels of type 1 inflammatory mediators.^50^ These results are important because microbial dysbiosis has been linked to asthma severity, treatment response, and exacerbation risk.^11^^,^^51^

##### Biologic-Treated Severe Asthma

Recent studies characterizing attacks in patients with severe asthma receiving biologic therapy have confirmed the heterogeneity of acute events in this population*.* The Mepolizumab Exacerbations (MEX) study, a multicenter, prospective, observational cohort study, was conducted in 140 patients treated with mepolizumab. Participants underwent clinical review before starting rescue treatment with OCSs or antibiotic therapy.^52^ The study identified 2 distinct inflammatory phenotypes of attacks based on sputum: eosinophilic (≥ 2% eosinophils) and noneosinophilic. Eosinophilic attacks (48% of the cohort) were characterized by significantly higher Feno (57 ppb vs 24 ppb), elevated BEC (70 cells/μL vs 30 cells/μL), and worse FEV_1_ % predicted (56% vs 72%). Conversely, noneosinophilic attacks occurred in 48% of patients and were associated with significantly higher C-reactive protein concentrations (15 mg/L vs 2 mg/L) and sputum neutrophils (90% vs 37%) and more frequently were treated with antibiotics (50% vs 19%), reflecting an infectious cause.^52^ Feno showed high diagnostic accuracy in identifying eosinophilic exacerbations occurring in patients receiving mepolizumab.

Building on these findings, an unsupervised clustering of sputum proteomic data identified 2 distinct subgroups within the MEX cohort. Cluster 1 comprised patients with persistently elevated airway inflammation despite mepolizumab treatment, driven by neutrophilic activation and epithelial signaling and with worse lung function and asthma control, suggesting resistance to treatment. Cluster 2 showed reduced airway inflammation, even when clinically stable, and reactivation at exacerbation, reflecting a more responsive inflammatory profile.^53^

Diver et al^54^ conducted an exploratory analysis of sputum samples collected in the MEX study combined with samples from the Refractory Asthma Stratification Program study. Patients with high Feno (≥ 50 ppb), when stable and at exacerbation, exhibited less dispersed microbial profiles, higher α diversity, and a lower mean relative abundance of *Proteobacteria*, particularly *H influenzae*. In contrast, low and mid Feno subgroups showed greater microbial heterogeneity, with approximately 20% of patients displaying a *Proteobacteria*-dominant profile, a finding associated with neutrophilic inflammation and increased infection risk. In a subset of 13 patients with paired sputum samples before initiation of mepolizumab and after ≥ 12 weeks of treatment, the biologic did not alter bacterial load or increase in *Proteobacteria* abundance.^54^

The Benralizumab Exacerbations study aimed to investigate the inflammatory phenotypes of attacks in patients treated with benralizumab, a monoclonal antibody targeting the IL-5 receptor. This multicenter observational study enrolled 157 patients with severe eosinophilic asthma, with 91 patients (58%) experiencing at least 1 exacerbation during the follow-up. During attacks on benralizumab, eosinophils remained suppressed in blood and sputum (median, 0 cells/μL). Despite this, Feno levels were elevated (median, 49 ppb). Increased sputum neutrophil percentages (67%) and elevated C-reactive protein (15 mg/L) also were found.^55^ These findings suggest that attacks in patients receiving benralizumab predominantly involve infective features, with Feno elevation potentially indicating noneosinophilic airway type 2 inflammation.^48^

Recently, the Mechanisms Underlying Asthma Exacerbations Prevented and Persistent with Immune Based Therapy: a Systems Approach Phase 2 (MUPPITS-2) trial demonstrated, through nasal transcriptomics, that despite the suppression of eosinophil-associated type 2 gene modules by mepolizumab, asthma exacerbations persisted and were driven by epithelial stress, innate immune responses, and mucus secretion. Feno levels remained elevated during illness and were associated with both type 2 and epithelial activation signatures, suggesting a broader inflammatory role.^56^

Finally, Howell et al^57^ conducted the Breakthrough Asthma Attacks Treated With Oral Steroids study, a prospective observational study involving 60 patients experiencing asthma attacks, with 56% receiving anti-IL-5 therapy and 44% receiving anti-IL-5R therapy. The predefined characterization of attacks according to Feno (Feno ≥ 25 ppb vs < 25 ppb)^58^ largely confirmed the observations made in the MEX study, whereby Feno-low attacks included greater proportions of patients with viral infections and were difficult to differentiate from Feno-high events on clinical evaluation. Biomarker-stratified outcomes after OCS treatment in the Breakthrough Asthma Attacks Treated With Oral Steroids study are discussed in the section entitled Emerging Strategies: Precision Medicine in Action.

##### What Endotypes Tell Us

Collectively, these studies highlight the need for biomarker measurement beyond clinical or functional assessments during asthma attacks, complementing chronic severe asthma phenotyping. Importantly, the underlying pathobiological characteristics are dynamic, influenced by the episode’s cause and the patient’s treatment regimen.

### Management of Asthma Attacks: From Current to Emerging Strategies

#### Current Approaches

##### OCSs, Antibiotics, or Both

The management of asthma attacks largely has relied on OCS treatment as the first-line treatment for the last 70 years*.*^59^ The evidence for this approach was summarized in 2 Cochrane systematic reviews that showed that OCS treatment reduces admission rates by 50% and the rate of relapse at 21 days by 53%.^60^^,^^61^ However, these effects must be weighed against the risks of overuse of repeated courses of OCS treatment, including increasing all-cause mortality.^9^ Importantly, these meta-analyses comprised trials dating from before 1993 that included few patients using inhaled corticosteroids (approximately 20%) and lack of information on key outcomes such as symptoms and lung function. Furthermore, inflammatory biomarkers were not measured to stratify the response.^60^^*,*^^61^ It is unclear if these results would be replicated in the era of antiinflammatory reliever (AIR) therapy (discussed herein), or if they apply across biomarker-stratified populations. Antibiotics in asthma attacks continue to be overprescribed, with real-world evidence showing that 22% to 44% of patients are treated with antibiotics as well as OCS.^11^ Indeed, a Cochrane review showed that antibiotics given at the time of exacerbation did not improve outcomes such as symptoms, lung function, or hospital admission rates when compared with standard care or placebo.^62^

##### Antiinflammatory Reliever Therapy

**Figure 1:**
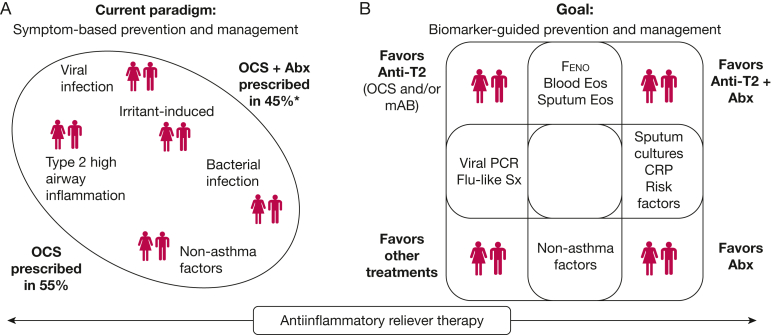
Diagrams showing 2 different approaches to asthma attacks. A, In the current paradigm for symptom-based management of asthma, we prevent and treat all acute events equally or randomly, at least with OCS and often with Abx treatment. B, Our goal is to inform both our preventive and therapeutic decisions better with objective evidence of the underlying biological characteristics using accessible biomarkers, many of which are available as point-of-care tests. These may enable more targeted or novel use of type 2 targeting antiinflammatory therapy with OCS, an mAb, or both. All attacks should benefit from antiinflammatory reliever therapy. Abx = antibiotics; CRP = C-reactive protein; Eos = eosinophil; Feno = fractional exhaled nitric oxide; mAb = monoclonal antibody; OCS = oral corticosteroid; PCR = polymerase chain-reaction; Sx = symptoms. ∗Data reported by Murray et al^71^ in (A); figure modified with permission from Ramakrishnan and Couillard.^11^

Historically, short-acting β_2_-agonists have provided symptomatic relief. However, they do not address the underlying airway inflammation, and their overuse may increase the severity and frequency of attacks.^63^ Interestingly, approximately 2 weeks before an asthma attack, rising airway inflammation may precede the decline in lung function*.*^64^ In turn, worsening symptoms may prompt patients to increase the use of reliever treatment. This window of opportunity presents a critical period for early intervention. Accordingly, GINA recommends AIR therapy, which involves low-dose inhaled corticosteroids plus formoterol or inhaled corticosteroids plus short-acting β_2_-agonists as needed for symptom control and exacerbation prevention.^1^^,^^65^ The evidence that supports this strategy recently was updated in a systematic review with network meta-analysis that included 27 randomized clinical trials (N = 50,496 adults and children). Compared with short-acting β_2_-agonists alone, AIR therapies were associated with a 16% to 35% reduction in severe attacks. Furthermore, AIR therapy was associated with improved asthma control and was not associated with increased risk of serious adverse events. In subgroup analyses, results were consistent along GINA steps 1 to 4.^66^ These results are confirmed in a Cochrane review.^67^ Overall, the benefits of using the AIR approach on attacks seem to be independent of biomarker (BEC and Feno) profile.^68^^,^^69^ Given its robust efficacy and safety profile, the AIR approach should be considered a standard of care for all patients with asthma, regardless of disease severity, as the updated British Thoracic Society guidelines recommend.^70^ The current approach to asthma attacks is shown in Figure 1.

#### Emerging Strategies: Precision Medicine in Action

##### Biomarker-Stratified Treatment Responses

Independent and complementary mechanistic information provided by clinically accessible biomarkers^11^^,^^48^ has enabled a refined understanding of the heterogeneity in acute treatment responses *(*Table 1*).* To explore the relationship between type 2 inflammatory biomarkers and treatment response to OCS treatment in people with asthma experiencing a severe attack, Celis-Preciado et al^47^ analyzed the PRISMA study cohort according to the predefined primary outcome, change in FEV_1_ after bronchodilation after a 7-day course of OCS treatment. The change in FEV_1_ after bronchodilation increased with combined BEC and Feno elevation, peaking in the type 2-high/high (BEC ≥ 300 cells/μL and Feno ≥ 35 ppb) phenotype (mean [SD], 0.390 [0.512] L). Conversely, type 2-low/low (BEC < 150 cells/μL and Feno < 25 ppb) attacks achieved nonsignificant FEV_1_ changes (mean [SD], 0.017 [0.153] L), albeit with high variability. Dual-biomarker (BEC plus Feno) stratification outperformed symptoms, radiography, microbiology, and FEV_1_-based parameters in univariable and multivariable models predicting lung function improvement. All patients showed improved Asthma Control Questionnaire scores, numerically peaking in the type 2-high/high phenotype (mean [SD], –1.58 [0.60]; *P* = .08). The groups experienced similar OCS-attributable adverse events, with 33 participants (62%) reporting ≥ 1 event.^72^ Together, these findings show that, as in chronic asthma, greater type 2 burden identifies a distinct lung function therapeutic trajectory, whereas OCS-related adverse events are distributed uniformly.

**Table 1:** Evidence Summary of Recent Key Studies of Asthma Attacks

Study (Year)	Type of Study	Population	Intervention	Results	Conclusions
PRISMA (2024)^47^	Observational prospective	53 patients GINA steps 1-5 (median age, 49 y; 60% female)	Prednisolone 40 mg/d for 7 d	• Attacks were classified according to an ordinal BEC and Feno 3-group categorization (type 2-low/low: BEC < 150 cells/μL and Feno < 25 ppb; type 2-high/high: BEC ≥ 300 cells/μL and Feno ≥ 35 ppb; and type 2-mid: not meeting other criteria).	• BEC and Feno stratify attacks independently of the presence of clinical features, confirming their complementary mechanistic value.
				• No differences between groups regarding baseline symptom scores, lung function, or frequency of infections. The change in FEV_1_ after bronchodilation with prednisone increased with combined BEC and Feno elevation, peaking in the type 2-high/high phenotype (mean [SD], 0.390 [0.512] L). Type 2-low/low attacks achieved nonsignificant FEV_1_ changes (mean [SD], 0.017 [0.153] L).	• Greater type 2 burden identifies a distinct lung function therapeutic trajectory, whereas OCS-related adverse events are distributed uniformly.
				• All patients showed improved ACQ scores, numerically peaking in the type 2-high/high phenotype (mean [SD], –1.58 [0.60]; *P* = .08).	
				• The groups experienced similar OCS-attributable adverse events (62%).	
RASP (2022)^49^	Subanalysis of a randomized controlled trial	183 patients GINA steps 4-5 (median age, 55 y; 71% female)	None	• Attacks were classified as type 2-low (Feno ≤ 20 ppb and BEC ≤ 150 cells/μL; 24%) and type 2-high (Feno > 20 ppb or BEC > 150; 76%).	Asthma exacerbations demonstrating a type 2-low phenotype are physiologically and symptomatically like type 2-high exacerbations.
				• At exacerbation, the type 2-low events were indistinguishable from type 2-high exacerbations in terms of lung function (mean [SD] fall in type 2-low FEV_1_, 200 [400] mL vs type 2-high 200 [300] mL; *P* = .93) and symptom increase (ACQ-5: type 2-low, 1.4 [0.8] vs type 2-high, 1.3 [0.8]; *P* = .72), with no increase in type 2 biomarkers from stable to exacerbation state in the type 2-low exacerbations.	
MEX (2021)^52^	Observational prospective	140 patients with severe asthma (median age, 55 y; 48% female) receiving treatment with mepolizumab	None	• Attacks were classified according to eosinophilic (sputum Eos ≥ 2%) or noneosinophilic (< 2%) exacerbations	Exacerbations with mepolizumab are 2 distinct inflammatory phenotypes, which largely can be differentiated using Feno: noneosinophilic events are driven by infection with a low Feno and high CRP, whereas eosinophilic exacerbations show a high Feno.
				• Eosinophilic exacerbations were characterized by higher Feno (57 ppb vs 24 ppb; *P* = .0004), elevated BEC (70 cells/μL vs 30 cells/μL; *P* = .0009), and lower FEV_1_ % predicted (56% predicted vs 72% predicted; *P* = .0075).	
				• Noneosinophilic exacerbations occurred in 48% of patients and were associated with elevated CRP (15 mg/L vs 2 mg/L; *P* < .0001) and higher sputum neutrophils (90% vs 37%; *P* < .0001) and were treated more frequently with antibiotics (50% vs 19%; *P* = .031), reflecting an infectious cause.	
				• Feno ≤ 20 ppb showed an NPV of 100% (95% CI, 80%-100%) and Feno ≥ 50 ppb showed a PPV of 77% (95% CI, 60%-90%) for eosinophilic exacerbations.	
MEX (2025)^53^	Translational substudy	43 patients enrolled in the MEX cohort with sputum samples collected: before mepolizumab (n = 28), stable with mepolizumab (n = 43), and at exacerbation (n = 26)	None	• Two inflammatory clusters were identified through unsupervised sputum proteomic analysis.	A subgroup of patients with increased neutrophilic activation, epithelial signaling, and microbiome alteration show less favorable clinical outcomes with anti-IL-5 biologic therapy.
				• Cluster 1 (nonresponders): persistent airway inflammation despite mepolizumab, with sustained elevation of IL-1β, IL-6, TSLP, MPO, NETs, and mucins; higher ACQ-5; lower FEV_1_; longer asthma duration; and increased bacterial load.	
				• Cluster 2 (responders): reduced airway inflammation while stable with mepolizumab, with reactivation during exacerbation.	
				• Both clusters showed similar Feno and blood eosinophils.	
BenRex (2024)^55^	Observational prospective	157 patients with severe asthma (median age, 55 y; 56% female) receiving treatment with benralizumab	None	• 91 patients (58%) experienced an exacerbation during the 12-18-month follow-up.	• During exacerbations with benralizumab, eosinophils remain suppressed in blood and sputum; elevated infective features such as sputum neutrophilia and rise in CRP occur.
				• At exacerbation, median for BEC (× 10^9^/L) and sputum Eos was 0% [IQR, 0%-0%], and Feno was 49 ppb [IQR, 29-92 ppb]. Median sputum neutrophil was 66% [IQR, 34%-84%] and mean (SD) CRP was 14.6 (23) mg/L.	• Feno remains elevated, potentially indicating noneosinophilic airway type 2 inflammation.
BOOST (2024)^57^	Observational prospective	60 patients with severe asthma (median age, 56 y; 63% female) receiving treatment with anti-IL-5/IL-5R biologics	Prednisolone 40 mg/d for 7 d	• Attacks were classified as Feno-high (≥ 25 ppb) or Feno-low (< 25 ppb).	Feno testing at attack can identify patients receiving anti-IL-5/IL-5R treatment who receive the most clinical benefit from prednisolone.
				• Primary outcome of 28-d treatment failure was similar for both groups.	
				• Patients with Feno-low attacks showed significantly higher likelihood of treatment failure at day 14 compared with those with Feno-high attacks (OR, 5.1; *P* = .02, post hoc analysis).	
				• At day 7, patients with Feno-high attacks showed significantly greater improvement in FEV_1_ (0.361 L; *P* = .02) and ACQ (–1.44; *P* < .001).	
ABRA (2024)^73^	Randomized controlled trial	158 patients with asthma (53%), COPD (32%), or both (12%; median age, 57 y; 54% female)	Benralizumab plus prednisone group (100 mg benralizumab subcutaneously once plus OCS for 5 d), benralizumab group (benralizumab plus placebo tablets), or prednisone group (placebo injection plus OCS)	• At 90 d, treatment failure occurred in 74% in the prednisone group and 45% in the pooled benralizumab group (OR, 0.26 [95% CI, 0.13-0.56]; *P* = .0005). The number needed to treat with pooled benralizumab to prevent a treatment failure was 4.	Benralizumab can be used as a treatment for acute eosinophilic exacerbations of asthma and achieves better outcomes than the current standard of care with prednisolone alone.
				• The 28-d total VAS symptoms scores were significantly better in the pooled benralizumab group compared with the prednisone group (mean difference, 49 mm [95% CI, 14-84 mm]; *P* = .0065).	
				• At day 28, a significant and clinically meaningful improvement in ACQ-7 and AQLQ was noted in the pooled benralizumab group compared with the prednisone group.	
				• Lung function parameters improved across all treatment groups with no difference between the prednisone and pooled benralizumab groups.	

ABRA = Acute Attacks Treated With Benralizumab; ACQ = Asthma Control Questionnaire; ACQ-5 = Asthma Control Questionnaire 5; ACQ-7 = Asthma Control Questionnaire 7; AQLQ = Asthma Quality of Life Questionnaire; BEC = blood eosinophil count; BenRex = Benralizumab Exacerbations; BOOST = Breakthrough Asthma Attacks Treated With Oral Steroids; CRP = C-reactive protein; Eos = eosinophil; Feno = fractional exhaled nitric oxide; GINA = Global Initiative for Asthma; IQR = interquartile range; MEX = Mepolizumab Exacerbations; MPO = myeloperoxidase; NET = neutrophil extracellular trap; NPV = negative predictive value; OCS = oral corticosteroid; ppb = parts per billion; PPV = positive predictive value; PRISMA = Phenotyping the Responses to Systemic Corticosteroids in the Management of Asthma Attacks; RASP = Refractory Asthma Stratification Program; TSLP = thymic stromal lymphopoietin; VAS = visual analog scale.

From an infectious perspective, the post hoc analysis^74^ of the Routine Molecular Point-of-Care Testing for Respiratory Viruses in Adults Presenting to Hospital With Acute Respiratory Illness (ResPOC) study^75^ in emergency or hospitalized patients with exacerbation of asthma or COPD is interesting. Although the original study did not show reduced antibiotic use with molecular testing, this subanalysis confined to participants with exacerbated airway diseases showed that the group with positive molecular findings for viruses had discontinued antibiotics earlier than those without infection.^74^ These results suggest that molecular testing may be relevant to direct antibiotic use in airway attacks. However, it is important to acknowledge that access to rapid molecular diagnostics is limited in many clinical settings, and their integration into routine care should be adapted to local resource availability.

For patients assessed with biologics, treatment response analysis by Howell et al^58^ of the Breakthrough Asthma Attacks Treated With Oral Steroids cohort is important. After clinical evaluation, 60 patients treated with anti-IL-5/IL-5R were treated with 40 mg prednisolone for 7 days. Prespecified groups were split according to Feno values measured at the time of the asthma attack, using a threshold of < 25 ppb or ≥ 25 ppb (Feno-high). Although the primary outcome of 28-day relapse after prednisolone treatment was similar for both groups, the patients with Feno-low attacks showed a significantly higher likelihood of treatment failure at day 14 compared with the patients with Feno-high attacks (post hoc analysis). For secondary outcomes on day 7, patients with Feno-high attacks showed significantly greater improvement in FEV_1_ (0.361 L) and Asthma Control Questionnaire score (–1.44) compared with patients with Feno-low attacks.^57^ These findings confirm and extend those made in the PRISMA study, in which type 2 biomarker elevation is associated with distinct therapeutic responses after OCS prescription.

In summary, significant heterogeneity in OCS benefits has been observed in asthma attacks occurring with and without biologics. This heterogeneity can be identified and predicted only through incorporation of type 2 biomarkers (BEC and Feno) into clinical assessment.

##### Acute Biologics for Asthma Attacks: Finally Here?

Biologics have emerged as a transformative approach in the chronic management of severe asthma, particularly for diseases driven by underlying type 2 inflammation.^76^^,^^77^ Recently, a landmark study explored the potential for benralizumab to treat eosinophilic airway attacks.^73^ Ramakrishnan et al^73^ conducted the Acute Attacks Treated With Benralizumab randomized placebo-controlled trial. This phase 2 study included 158 adult patients with a confirmed exacerbation of asthma or COPD and BEC of ≥ 300 cells/μL. Participants were randomized into 3 groups: 100 mg benralizumab subcutaneously once plus OCS for 5 days, benralizumab plus placebo tablets, or placebo injection plus OCS. The time to the first treatment failure event was significantly longer in the pooled benralizumab plus placebo tablets group compared with the placebo injection plus OCS group. The number needed to treat with pooled benralizumab plus placebo tablets to prevent a treatment failure was 4. The 28-day total visual analog scale symptoms score also was significantly better in the pooled benralizumab plus placebo tablets group compared with the placebo injection plus OCS group. This trial marks the first novel therapeutic for airway attacks in the last half-century.

A major strength of the trial was its endotype-driven approach, which selected patients with eosinophilic asthma, COPD, or both. A key limitation was that only 1 in 8 patients experiencing attacks were recruited from the emergency department, potentially underrepresenting the most severe attacks, typically requiring hospital admission. Additionally, the study was not powered to differentiate outcomes between patients with asthma attacks and those with COPD attacks. Finally, a trend of greater improvement with 100 mg benralizumab subcutaneously once plus OCS for 5 days in the first 2 weeks may warrant further exploration. Nevertheless, the Acute Attacks Treated With Benralizumab study paves the way to acute precision medicine approaches, moving away from the conventional one-size-fits-all OCS strategy.

## Future Directions

**Figure 2:**
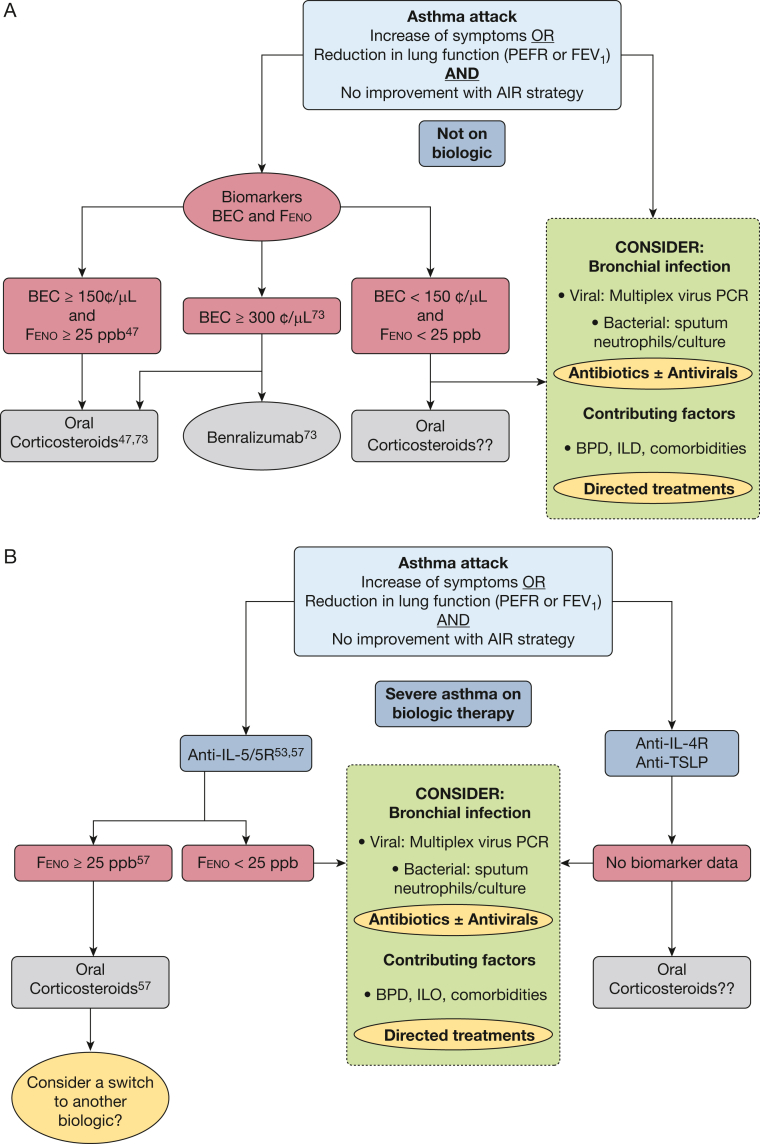
Diagrams showing biomarker-guided management approach for asthma attacks. Asthma attacks involve an increase in symptoms or a reduction in lung function, with no improvement despite an antiinflammatory reliever. The figure presents a proposed clinical algorithm for managing asthma attacks based on BEC and Feno, as supported by recent evidence. A, Stratification for patients not receiving biologic therapy. Those with elevated type 2 biomarkers (BEC ≥ 150 cells/μL and Feno ≥ 25 parts per billion [ppb] or BEC ≥ 300 cells/μL) are considered for OCS treatment (Phenotyping the Responses to Systemic Corticosteroids in the Management of Asthma Attacks and Acute Attacks Treated With Benralizumab [ABRA] studies),^45^^,^^75^ with the potential use of benralizumab in selected patients (ABRA study).^75^ It is uncertain whether patients with low type 2 biomarkers (BEC < 150 cells/μL and Feno < 25 ppb) could benefit from OCS treatment and should be evaluated for viral or bacterial infection or alternative symptom contributors. However, access to rapid-turnaround microbiological testing may be limited in some clinical settings and should be considered in the context of local resource availability. B, Approach for patients with severe asthma who are already receiving biologics. In patients receiving mepolizumab, benralizumab, or reslizumab, elevated Feno during the attack identifies patients who benefit from OCS treatment (Mepolizumab Exacerbations study and the Breakthrough Asthma Attacks Treated With Oral Steroids study),^50^^,^^53^ and switching biologic therapy could be considered. For patients with low Feno, OCS treatment in this group remain individualized, and infection and comorbid conditions should be evaluated and treatments directed accordingly. In patients receiving treatment with dupilumab or tezepelumab, data on biomarkers to guide OCS treatment in asthma attacks are limited. AIR = antiinflammatory reliever; BEC = blood eosinophil count; BPD = breathing pattern disorder; Feno = fractional exhaled nitric oxide; ILO = inducible laryngeal obstruction; PEFR = peak expiratory flow rate; OCS = oral corticosteroid; PCR = polymerase chain reaction; TSLP = thymic stromal lymphopoietin.

The evolving understanding of the heterogeneity of asthma attacks, driven by advances in immunology and clinical research, underscores the need for a paradigm shift toward precision medicine. Several priority areas for future research and clinical application have emerged, focusing on refining definitions of asthma exacerbations, enhancing identification through biomarkers, and developing personalized therapeutic strategies (Table 2). A precision medicine-based approach to asthma attacks, shown in Figure 2, requires validation.

**Table 2:** Future Research Questions in Asthma Attacks

Area of Research	Key Questions
Definition and classification	• How can asthma attack definitions be refined to include biomarker-guided criteria?
	• How can patient-reported outcomes be standardized to improve attack classification?
	• What severity thresholds should be used for clinical and research purposes?
Incorporation of biomarkers	• What combinations of biomarkers offer the highest predictive value for treatment response?
	• Can real-time point-of-care biomarker testing improve treatment decisions?
	• What is the role of BEC, Feno, or both in asthma attacks in patients receiving dupilumab or tezepelumab?
Biological mechanisms and microbiome	• What are the immunologic drivers of non-type 2 inflammation during asthma attacks?
	• What biomarkers for non-type 2 pathways could be harnessed to develop novel targeted therapies?
	• How does microbial dysbiosis impact asthma attack severity and treatment outcomes?
	• What is the relationship between airway microbiome diversity and steroid response?
	• Can single-cell transcriptomics uncover novel asthma endotypes linked to exacerbations?
Treatment	• What is the role of OCS therapy, AIR therapy, or both in type 2-low asthma attacks?
	• How can biologics be tailored to specific asthma phenotypes?
	• What biomarkers differentiate treatment responders from nonresponders during exacerbations?
Health economics and policy	• What are the health economic implications of routine biomarker-guided care?
	• What are the health economic implications of biologic use in acute asthma?
	• What incentives can encourage health care providers to adopt personalized asthma care?

AIR = antiinflammatory reliever; BEC = blood eosinophil count; Feno = fractional exhaled nitric oxide; OCS = oral corticosteroid.

## Summary

Asthma attacks are the cause of substantial morbidity, health care use, and avoidable deaths.^78^ In effect, attacks are red flags^79^: a sentinel event, but also an opportunity for effective, personalized management of asthma.^27^ Despite the evident heterogeneity of mechanisms driving asthma attacks, the standard of care in acute asthma has remained unchanged for 70 years and consists of a 1-size-fits-all treatment with OCSs and often antibiotics.^11^^,^^59^ Based on the available literature, novel approaches applying precision medicine strategies for exacerbation management warrant further research (Figs 1, 2).

## Funding/Support

S. C. was supported by the Association Pulmonaire du Québec’s Research Chair in Respiratory Medicine and the 10.13039/501100000156Fonds de Recherche du Québec - Sante. C. C.-P. was supported by the Université de Sherbrooke graduate scholarships, Canadian Allergy, Asthma, and Immunology Foundation (CAAIF) and Fonds de Recherche du Québec – Santé (FRQS) scholarship.

## Financial/Nonfinancial Disclosures

The authors have reported to *CHEST* the following: C. C.-P. has received speaker honoraria from AstraZeneca and Sanofi-Regeneron and consultancy fees from AstraZeneca and Sanofi-Regeneron. S. R. reports speaker honoraria from GSK, AstraZeneca, Sanofi, and Boehringer Ingelheim, unrestricted research grants from AstraZeneca to his institution, and conference travel support from AstraZeneca and Boehringer Ingelheim. I. H. reports conference travel from GSK, an National Institute for Health and Care Research (NIHR) Biomedical Research Centre grant, a grant from the British Medical Association Foundation. M. E. W. has received consulting, advisory, or speaking honoraria from the following: Allakos, Amgen, Areteia Therapeutics, Arrowhead Pharmaceutical, AstraZeneca, Avalo Therapeutics, Belenos Bio, Celldex, Connect Biopharma, Eli Lilly, Equillium, Glaxosmithkline, Incyte, Jasper Therapeutics, Kinaset, Kymera, Merck, MyBiometry, Pharming, Phylaxis, Pulmatrix, Rapt Therapeutics, recludix Pharma, Regeneron, Roche/Genentech, Sanofi/Genzyme, Sentien, Sound Biologics, Tetherex Pharmaceuticals, Uniquity Bio, Upstream Bio, Verona Pharma, and Zurabio. P. A. reports consulting fees from AstraZeneca, GlaxoSmithKline, Connect Biopharma, Amgen, and Sanofi/Genzyme, as well as honoraria from AstraZeneca and Sanofi/Genzyme. S. C. reports nonrestricted research grants from the NIHR Oxford Biomedical Research Centre, the Quebec Respiratory Health Research Network, the Association Pulmonaire du Québec, the Academy of Medical Sciences, AstraZeneca, bioMérieux, Circassia Niox Group, and Sanofi-Genyme-Regeneron; speaker honoraria from AstraZeneca, GlaxoSmithKline, Sanofi-Regeneron, Circassia Niox Group, and Valeo Pharma; consultancy fees for FirstThought, Apogee Therapeutics, Upstream Bio, AstraZeneca, GlaxoSmithKline, Sanofi-Regeneron, Access Biotechnology, and Access Industries; sponsorship to attend or speak at international scientific meetings for AstraZeneca and Sanofi-Regeneron; serving as an advisory board member and owning stock options for Biometry, Inc., a company that is developing an Feno device (myBiometry); being a coinventor for the patent filed as “Method for Alleviating Dyspnea With Neuromodulation”; being an advisor to the Institut National d’Excellence en Santé et Services Sociaux for an update of the asthma general practice information booklet for general practitioners as well as therapeutic indications for Enerzair; and being a member of the asthma steering committee of the Canadian Thoracic Society. None declared (E. B. H. K.).
